# Mastozytose bei Kindern

**DOI:** 10.1007/s00105-023-05168-9

**Published:** 2023-05-04

**Authors:** Hanna Wassmer, Karin Hartmann

**Affiliations:** Allergologische Poliklinik, Klinik für Dermatologie, Universitätsspital Basel, Petersgraben 4, 4031 Basel, Schweiz

**Keywords:** Antihistaminika, *KIT*-Mutation, Mastozytose, Tryptase, Urticaria pigmentosa, Antihistamines, *KIT* mutation, Mastocytosis, Tryptase, Urticaria pigmentosa

## Abstract

Die Mastozytose bei Kindern ist eine seltene Erkrankung, die durch eine abnorme Vermehrung von Gewebemastzellen gekennzeichnet ist. Es zeigen sich typische Hautveränderungen, die als makulopapulöse kutane Mastozytose, diffuse kutane Mastozytose oder Mastozytom klassifiziert werden. Ein Teil der Patientinnen und Patienten weist zudem Mastzellmediatorsymptome wie Juckreiz, Flush und Anaphylaxie auf. Bei vielen Kindern ist die Erkrankung durch einen benignen, meist selbstlimitierenden Verlauf charakterisiert; nur selten findet sich eine systemische Mastozytose mit extrakutaner Beteiligung und chronischem oder progressivem Verlauf. Therapeutisch werden in erster Linie H_1_-Antihistaminika eingesetzt, je nach Schwere bedarfsorientiert oder als Dauertherapie. Kinder, Eltern und Betreuungspersonen sollten sorgfältig über das Krankheitsbild und mögliche Trigger-Faktoren der Mastzellmediatorfreisetzung aufgeklärt werden. Für Kinder mit ausgeprägten Hautveränderungen und schweren Symptomen ist die Verordnung eines Adrenalin-Autoinjektors zur Notfallbehandlung empfehlenswert.

## Abstract (en)

Mastocytosis in children is a rare disease characterized by an abnormal accumulation of tissue mast cells. Mastocytosis in children presents with typical skin alterations that are classified as maculopapular cutaneous mastocytosis, diffuse cutaneous mastocytosis, or mastocytoma. Some patients also develop mast cell mediator symptoms, such as pruritus, flush, and anaphylaxis. In many children, the disease is characterized by a benign and usually self-limiting course; systemic mastocytosis with extracutaneous involvement and a chronic or progressive course is found only rarely. Therapeutically, H1 antihistamines are primarily used on an as-needed basis or as continuous treatment, depending on the severity. Children, parents and caregivers should be thoroughly educated about the clinical picture and possible trigger factors of mast cell mediator release. For children with extensive skin alterations and severe symptoms, the prescription of an epinephrine auto-injector is recommended for emergency treatment.

## Ätiologie und Pathogenese

Kennzeichnend für die Mastozytose ist eine pathologische Vermehrung von Mastzellen in unterschiedlichen Geweben des Körpers [24]. Mastzellen sind Immunzellen, die aus Vorläuferzellen des Knochenmarks hervorgehen und in das Gewebe auswandern. Sie sind in besonders großer Anzahl in Haut und Schleimhäuten zu finden, und damit in Geweben, die direkt mit der Außenwelt in Verbindung stehen. Neben der Haut finden sich viele Mastzellen z. B. auch im Gastrointestinaltrakt und im Bronchialsystem. Mastzellen spielen eine zentrale Rolle bei Allergien und sind an der Regulation verschiedener Immunantworten beteiligt [15].

Bei der Mastozytose ist mindestens ein Organ von der Mastzellvermehrung betroffen. Am häufigsten sind Haut und Knochenmark beteiligt, aber auch Gastrointestinaltrakt, Milz, Leber und Lymphknoten können involviert sein. Die aktuelle WHO-Klassifikation der Mastozytosen, die für pädiatrische sowie adulte Patientinnen und Patienten gleichermaßen gilt, untergliedert zunächst in kutane und systemische Mastozytose sowie Mastzellsarkom (Tab. 1) [24, 25]. Die systemische Mastozytose wird nochmals in 6 verschiedene Formen unterteilt. Die weitaus häufigste Form der systemischen Mastozytose ist die indolente systemische Mastozytose. Die kutane Mastozytose ist durch die singuläre Beteiligung der Haut, und damit das Fehlen einer Beteiligung des Knochenmarks oder anderer extrakutaner Organe, definiert [10].

**Table 1:** 

Kategorien der Mastozytose	Typisches Vorkommen bei Kindern oder Erwachsenen
**Kutane Mastozytose**	
*Makulopapulöse kutane Mastozytose (früher bezeichnet als Urticaria pigmentosa)*	Kinder
Polymorphe makulopapulöse kutane Mastozytose	Kinder
Monomorphe makulopapulöse kutane Mastozytose	Erwachsene
*Diffuse kutane Mastozytose*	Kinder und Erwachsene
*Mastozytom*	Kinder
**Systemische Mastozytose**	
*Indolente systemische Mastozytose*	Erwachsene
*Knochenmarkmastozytose*	Erwachsene
*Schwelende systemische Mastozytose*	Erwachsene
*Aggressive systemische Mastozytose*	Erwachsene
*Systemische Mastozytose mit assoziierter hämatologischer Neoplasie*	Erwachsene
*Mastzellleukämie*	Erwachsene
**Mastzellsarkom**	Erwachsene

Die Mastozytose ist mit einer Prävalenz von etwa 1:10.000 eine seltene Erkrankung [3]. Man geht jedoch davon aus, dass die Mastozytose bisher noch unterdiagnostiziert ist und die Prävalenz möglicherweise höher als dieser Wert ist.

Es werden 65 % der Mastozytosediagnosen in der Kindheit gestellt. Bei einem Großteil der pädiatrischen Patientinnen und Patienten wird eine kutane Mastozytose mit benignem, selbstlimitierendem Verlauf beobachtet [11, 16]. In der Mehrzahl der Fälle zeigt sich eine spontane Remission bis zum Erreichen der Adoleszenz [27]. Bei Persistieren der Hautveränderungen bis ins Erwachsenenalter ist dagegen der Nachweis einer systemischen Mastozytose mit Knochenmarkbeteiligung möglich [3, 10].

Die Mastozytose ist in der frühen Kindheit beim männlichen Geschlecht etwas häufiger zu finden, während im Erwachsenenalter ein leicht vermehrtes Auftreten beim weiblichen Geschlecht zu beobachten ist [17, 19, 23].

Die Mastozytose ist eine klonale Erkrankung, die meist mit sporadischen, aktivierenden Mutationen im Protoonkogen *KIT* verbunden ist. Während bei mehr als 80 % der adulten Patientinnen und Patienten die somatische Punktmutation *KIT* D816V in Exon 17 zu finden ist [2], zeigen Kinder mit Mastozytose nur z. T. die Mutation *KIT* D816V, z. T. aber auch andere Mutationen des *KIT*-Gens oder keine KIT-Mutation [16]. Mithilfe der sensitiven Polymerase-Kettenreaktion (PCR) lässt sich inzwischen die Allellast der *KIT*-D816V-Mutation auch im peripheren Blut bestimmen. Eine kürzlich publizierte Studie konnte zeigen, dass die *KIT*-D816V-Allellast im peripheren Blut bei Kindern mit milder kutaner Mastozytose oft unterhalb der Nachweisgrenze lag, während eine Systembeteiligung wahrscheinlich war, wenn die *KIT*-D816V-Mutation im peripheren Blut nachgewiesen wurde [5]. Die Mutationen des *KIT*-Gens führen zu einer ligandunabhängigen Aktivierung der Rezeptor-Tyrosinkinase KIT (CD 117). Unter physiologischen Bedingungen wird KIT durch den Liganden Stammzellfaktor (SCF) aktiviert und steuert die Differenzierung von myeloischen CD34-positiven Progenitorzellen aus dem Knochenmark sowie die Proliferation, Apoptose und Migration der Mastzellen. Diese Autoaktivierung des Rezeptors KIT trägt folglich zur Akkumulation von Mastzellen in unterschiedlichen Organen des Körpers bei [24].

In seltenen Fällen wurde bei einigen Familien das Auftreten einer hereditären Mastozytose mit autosomal-dominantem Erbgang beobachtet. Die familiäre Mastozytose ist meist mit Keimbahnmutationen des *KIT*-Gens verbunden, die jedoch außerhalb von Exon 17 liegen [12, 16].

## Klinische Manifestationen

### Hautveränderungen

Da die Mastozytose bei mehr als 90 % der betroffenen Kinder in die Kategorie der kutanen Mastozytose fällt, wird im Folgenden auf die verschiedenen Formen der kutanen Mastozytose, die durch unterschiedliche Hautveränderungen gekennzeichnet sind, vertiefend eingegangen. Insgesamt sind die Hautläsionen bei der kindlichen Mastozytose heterogener als bei der Erwachsenen-Mastozytose [10]. Die Hautveränderungen der Mastozytose sind in ca. einem Viertel der Fälle bereits bei Geburt vorhanden. Bei weiteren mehr als 70 % der Kinder entstehen sie in den ersten 6 Lebensmonaten, manchmal noch bis zum 2. Lebensjahr, und dagegen nur sehr selten erst nach dem 2. Lebensjahr [19].

Das „Darier-Zeichen“ ist bei allen Formen der kutanen Mastozytose positiv

Die kutane Mastozytose wird in 3 Hauptformen unterteilt (Tab. 1). Bei allen Formen treten nach mechanischer Reizung der Hautläsionen Quaddeln auf – ein pathognomonisches Zeichen der Mastozytose-Hautläsionen, das als „Darier-Zeichen“ bezeichnet wird.

#### Makulopapulöse kutane Mastozytose

Die häufigste Form der kutanen Mastozytose ist die makulopapulöse kutane Mastozytose. Sie wurde früher auch als Urticaria pigmentosa bezeichnet. Die makulopapulöse kutane Mastozytose wird nochmals in 2 Subgruppen unterteilt [10, 27].

##### Monomorphe makulopapulöse kutane Mastozytose.

**Figure 1:**
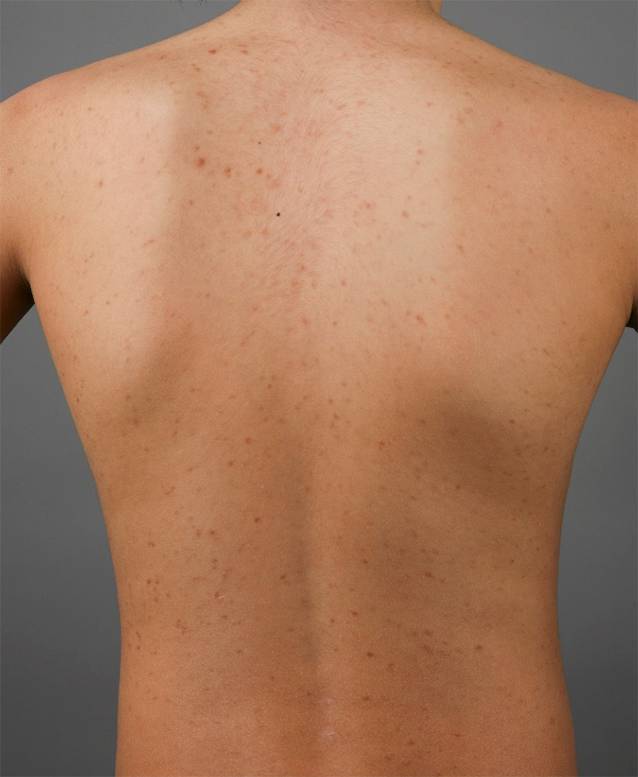

Monomorphe makulopapulöse Hautläsionen finden sich v. a. bei Erwachsenen. Ein Auftreten im Kindesalter ist jedoch möglich und betrifft meist die seltenen Fälle mit Beginn der Erkrankung erst nach dem 2. Lebensjahr und Persistenz der Hautläsionen bis ins Erwachsenenalter. Klinisch imponieren bei der monomorphen makulopapulösen Hautbeteiligung symmetrisch über den Körper verteilte, runde, kleine (bis zu 10 mm große), braun-rote makulopapulöse Läsionen (Abb. 1). Sie zeigen sich zu Beginn meist im Bereich der Oberschenkel und am Stamm lokalisiert und können im Verlauf auch am Hals und an den distalen Extremitäten auftreten. Das Gesicht, die Handflächen und die Fußsohlen bleiben typischerweise ausgespart. In seltenen Fällen finden sich bei dieser Form auch konfluierende Läsionen, die große Areale des Integuments betreffen können.

##### Polymorphe makulopapulöse kutane Mastozytose.

**Figure 2:**
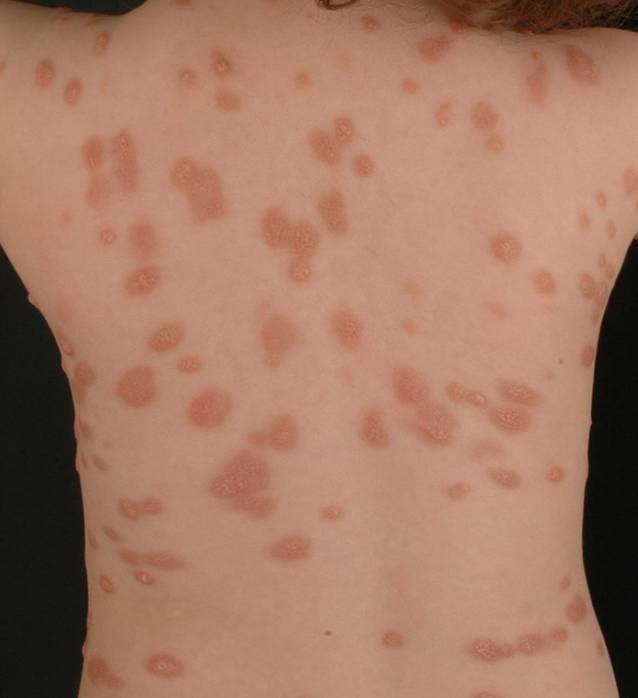

Bei den meisten betroffenen Kindern ist die polymorphe makulopapulöse kutane Mastozytose zu verzeichnen; diese ist durch unterschiedlich große, bis zu mehrere Zentimeter im Durchmesser messende, braune, rote oder gelbliche Läsionen gekennzeichnet (Abb. 2). Die Hautveränderungen können scharf oder unscharf begrenzt sein und sowohl nodulär, als Plaque oder auch flach in Erscheinung treten. Oft finden sind anfangs noduläre oder plaqueförmige Hautläsionen, die im Verlauf der Zeit abflachen. Meist sind die Hautveränderungen am Stamm und an den Extremitäten lokalisiert. Die Verteilung der polymorphen Läsionen ist typischerweise asymmetrisch. Ein besonderes Merkmal der polymorphen makulopapulösen kutanen Mastozytose ist, dass sich auch im Bereich der Schläfen und des behaarten Kopfes Mastozytoseläsionen finden. Durch mechanische Irritation ist, insbesondere in den ersten Lebensjahren, eine Blasenbildung im Bereich der Läsionen möglich [9, 10]. Im Rahmen einer Studie an 122 Patientinnen und Patienten mit makulopapulöser kutaner Beteiligung, die in der Kindheit begonnen hatte, konnten Wiechers et al. bei Patienten mit polymorphen Hautveränderungen im Vergleich zu den Patienten mit monomorphen Läsionen häufiger eine spontane Remission der Hautveränderungen, eine kürzere Erkrankungsdauer und niedrigere Tryptaselevel im Serum beobachten [27].

#### Diffuse kutane Mastozytose

**Figure 3:**
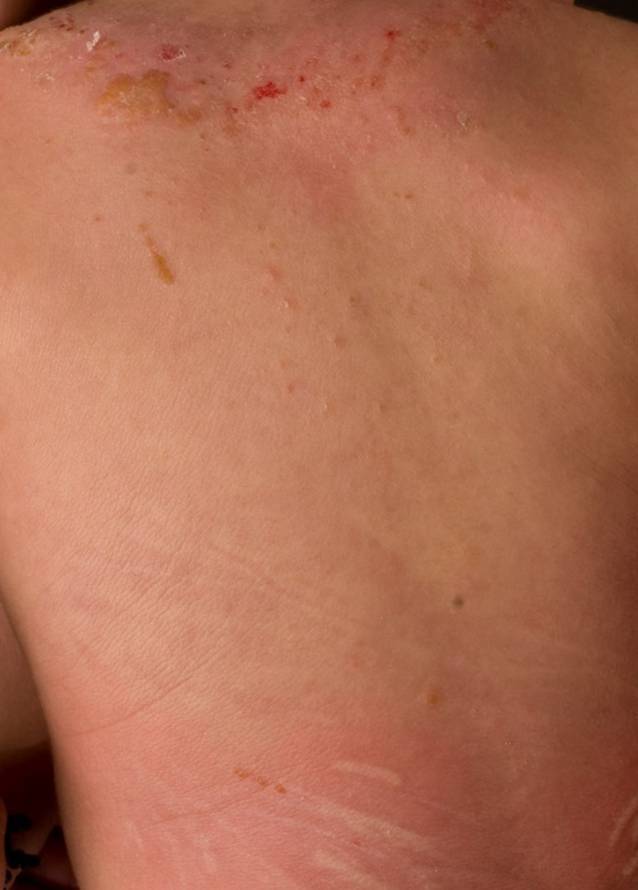

Als seltenere Form ist die diffuse kutane Mastozytosebeteiligung in der frühen Kindheit bei 5–13 % der pädiatrischen Patientinnen und Patienten zu finden [10, 16]. Die diffuse kutane Beteiligung ist durch eine homogene orangefarbene Hyperpigmentierung und Verdickung der gesamten Haut charakterisiert (Abb. 3). Anfangs besteht z. T. eine deutliche Erythrodermie. Bei mechanischer Reizung kommt es zu urtikariellem Dermografismus (Darier-Zeichen), aber auch zu großflächigen Quaddeln und z. T. Blasen. Insgesamt besteht bei der diffusen Form eine ausgeprägte Neigung zur Blasenbildung. Der Blaseninhalt ist meist klar, manchmal auch hämorrhagisch. Die Blasen heilen ohne Narbenbildung ab. Nach den ersten 2 bis 3 Lebensjahren gehen die Blasen zurück. Die basale Serumkonzentration der Tryptase ist bei diesen Patientinnen und Patienten anfangs häufig erhöht. Über mehrere Jahre fallen die Tryptasewerte langsam ab [27]. Auch sind systemische Beschwerden mit Flushing und Hypotension bis hin zu Anaphylaxien beschrieben. Die diffuse kutane Beteiligung kann außerdem hinweisend auf eine familiäre Mastozytose sein [12, 26]. Die erhöhten Tryptasewerte sind dann relativ stabil; es zeigt sich kein Rückgang der Tryptaselevel. Wie oben erwähnt, geht die familiäre Mastozytose häufig mit *KIT*-Keimbahnmutationen außerhalb von Exon 17 einher [12, 26]. Zum  Teil ist die familiäre Mastozytose mit gastrointestinalen Stromatumoren (GIST) assoziiert [12]. In einem Bericht wurde auch eine Verbindung mit tuberöser Sklerose beschrieben [21].

#### Mastozytom

**Figure 4:**
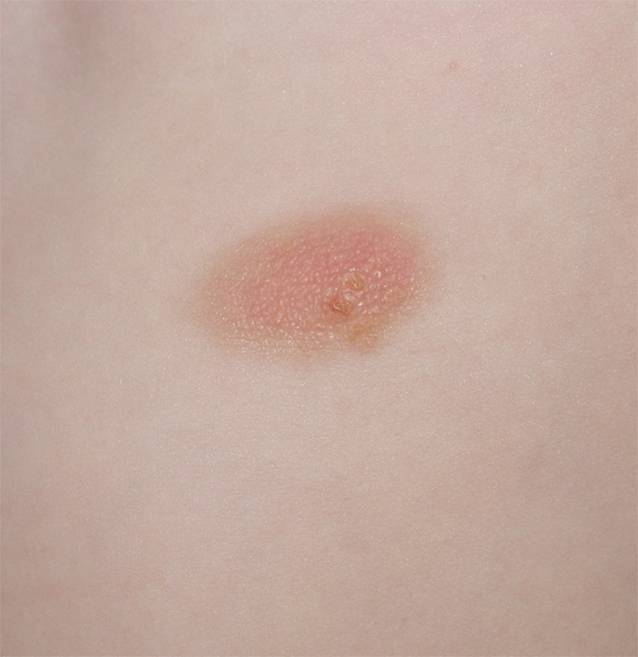

Das kutane Mastozytom tritt bei etwa 20 % der Kinder auf. Bei dieser Form findet sich nur eine Läsion, in Ausnahmefällen bestehen 2 oder 3 Hautveränderungen (Abb. 4). Die Läsionen sind rot oder braun, treten als Nodus, Plaque oder Makula auf und können eine Größe bis zu 10 cm erreichen. Mastozytome sind in den ersten Jahren oft mit Blasenbildung verbunden, insbesondere nach Kratzen und mechanischer Irritation. Häufig sind Mastozytome am Stamm lokalisiert, prinzipiell können sie aber ebenso an anderen Körperstellen auftreten, wie Handinnenflächen, Fußsohlen und am behaarten Kopf [10, 19].

### Mastzellmediatorsymptome

Verschiedene Trigger-Faktoren, z. B. mechanische Reize wie Reibung und Druck, schnelle Temperaturwechsel, Hitze, Kälte, körperliche Anstrengung, emotionaler Stress, Impfungen, Infektionen (v. a. durch Viren, seltener auch Bakterien oder Parasiten), Insektengifte, Narkosen und Medikamente, können die Freisetzung von Mediatoren aus Mastzellen induzieren. Zu diesen Mastzellmediatoren zählen Histamin, Proteasen (Tryptase, Chymase, Carboxypeptidase) und Heparin sowie sekundär durch Mastzellaktivierung gebildete Lipide wie Prostaglandin D_2_ und Leukotrien C_4_ [4, 15].

Bei Kindern mit einer Mastozytose führt die verstärkte Freisetzung von Mastzellmediatoren v. a. zu Juckreiz, der bei bis zu 79 % der Kinder beobachtet wird [4]. Des Weiteren sind das Auftreten von Quaddeln und Blasen im Bereich der Mastozytoseläsionen sowie generalisiertes Flushing mit plötzlich auftretenden Erythemen, v. a. im Gesicht und am Oberkörper, bis hin zu Hypotonien möglich. Insgesamt haben jedoch die meisten Kinder mit einer Mastozytose eine milde Beschwerdesymptomatik. Im Vergleich zur adulten Mastozytose sind Anaphylaxien deutlich seltener; sie werden bei weniger als 10 % der kindlichen Mastozytosen beobachtet [4]. Wenn Anaphylaxien auftreten, betreffen diese meist Kinder mit erhöhter Tryptasekonzentration und großflächigem Hautbefall [1].

Im Vergleich zur adulten Mastozytose sind Anaphylaxien bei betroffenen Kindern deutlich seltener

Weitere Symptome in extrakutanen Organen sind bei der pädiatrischen kutanen Mastozytose nur selten anzutreffen und dagegen v. a. bei Systembeteiligung zu beobachten. Hierzu zählen gastrointestinale Beschwerden mit Übelkeit, Diarrhö und Bauchkrämpfen; aber auch gastrointestinale Ulzera sind beschrieben. Respiratorische Beschwerden wie Rhinorrhö oder Husten finden sich bei der Mastozytose kaum. Neurologische Symptome wie Konzentrationsschwäche, aggressives Verhalten, Ängstlichkeit, Depression und autistisches Verhalten wurden bei Mastozytose diskutiert, jedoch nicht bestätigt [4, 16].

## Diagnostisches Vorgehen

Der erste Schritt in der Diagnostik umfasst die sorgfältige Inspektion des gesamten Integuments und die Beschreibung der Hautveränderungen (Tab. 2). Bestätigend kann das Darier-Zeichen getestet werden. Durch mehrmaliges moderates Streichen einer Mastozytoseläsion mit dem Holzspatel kommt es innerhalb weniger Minuten zu Rötung, Schwellung und Juckreiz, die auf die Läsion oder die unmittelbare Umgebung der Läsion beschränkt bleiben. Zu beachten ist jedoch, dass das Darier-Zeichen bei Kindern mit Mastozytomen oder ausgeprägten Hautveränderungen immer nur sehr vorsichtig getestet werden sollte, da es infolge der mechanischen Reizung auch zur Triggerung eines Flush, einer Hypotonie oder Anaphylaxie kommen kann [10].

**Table 2:** 

*Majorkriterium*
Typische Hautläsionen der Mastozytose, assoziiert mit dem Darier-Zeichen
*Minorkriterien*
Erhöhte Anzahl von Mastzellen in einer Biopsie aus läsionaler Haut
Nachweis einer (aktivierenden) *KIT*-Mutation in läsionaler Haut

Finden sich typische Mastozytoseläsionen und das Darier-Zeichen, bedarf es bei der pädiatrischen Mastozytose keiner Probebiopsie. Nur bei unklarem Befund wird die Entnahme einer läsionalen Hautbiopsie empfohlen. Meist ist histologisch eine deutlich erhöhte Zahl von Mastzellen (ca. 4- bis 8fach) nachweisbar, jedoch ist die Mastzellzahl insgesamt relativ variabel und je nach Körperregion unterschiedlich. Zudem sind viele andere Hauterkrankungen mit erhöhten Mastzellzahlen assoziiert. Als immunhistochemischer Marker wird ein Anti-Tryptase-Antikörper für die Färbung der Mastzellen empfohlen. Ergänzend können Giemsa- und Toluidinblaufärbungen angewendet werden [10, 16]. Eine Probebiopsie sollte behutsam erfolgen, um eine Degranulation der Mastzellen zu verhindern. Das Lokalanästhetikum sollte zirkulär um den Entnahmeort injiziert werden.

Die Konzentration der Serinprotease Tryptase korreliert mit der Mastzellzahl im Körper

Ein sehr hilfreicher Biomarker in der Mastozytosediagnostik ist der Tryptasewert im Serum. Die Serinprotease Tryptase wird überwiegend von Mastzellen produziert und korreliert gut mit der Mastzellzahl im Körper. Die Bestimmung der Tryptase dient sowohl der initialen Diagnosestellung als auch der Beurteilung des Verlaufs der Mastozytose [22].

Bei Kindern mit kutanen Formen und geringen klinischen Symptomen ist die basale Konzentration der Tryptase meist im Referenzbereich [4, 27]. Innerhalb des Referenzbereichs kann sie jedoch etwas höher sein, z. B. 5–10 ng/ml, während gesunde Kinder dagegen meist einen Wert kleiner als 5 ng/ml aufweisen. Es ist zu empfehlen, bei milden Verlaufsformen den Tryptasewert nur im Rahmen einer anderen Blutabnahme mitzubestimmen, eine separate Blutabnahme zur Abklärung der Mastozytose ist meist nicht notwendig.

Kinder mit erhöhter Tryptasekonzentration, ausgeprägten Hautveränderungen oder Hinweis auf systemische Mastozytose erhalten eine jährliche Verlaufskontrolle mithilfe der Tryptasemessung sowie Bestimmungen des Blutbilds und der laborchemischen Serumparameter.

Im Rahmen jeder Konsultation ist neben der Inspektion des Integuments eine abdominelle Palpation zu empfehlen.

Ergibt sich der Verdacht auf eine Organomegalie, und/oder liegt ein hoher Tryptasewert und/oder ein auffälliges Blutbild vor, wird zur Abklärung einer Systembeteiligung eine Knochenmarkbiopsie empfohlen [16]. Hilfreich ist außerdem die Bestimmung der *KIT*-D816V-Allellast im peripheren Blut. Carter et al. haben gezeigt, dass eine Organomegalie bei Kindern mit kutanen Läsionen einen starken Hinweis für eine Systembeteiligung darstellt [6]. Auch bei Persistenz der Hautveränderungen bis ins Erwachsenenalter sollte ein entsprechendes Screening zur Abklärung einer Systembeteiligung erfolgen.

**Figure 5:**
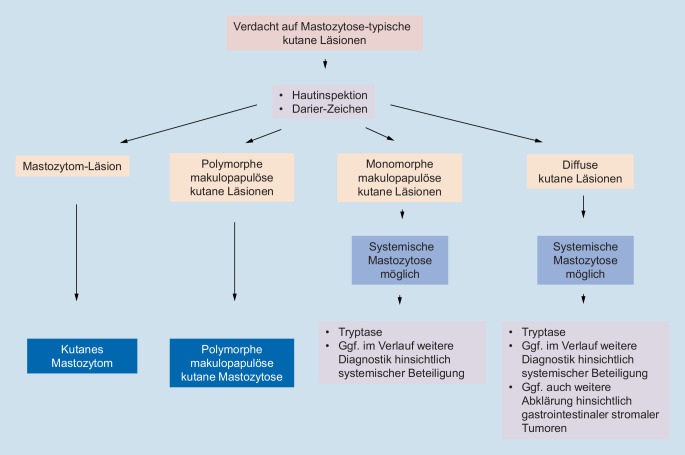

Ein praktischer diagnostischer Algorithmus zur Abklärung von mastozytosesuspekten Hautveränderungen bei Kindern findet sich in Abb. 5.

## Therapie

Eine kurative medikamentöse Therapie der Mastozytose ist nicht verfügbar. Es wird daher insgesamt eine symptomatische Behandlungsstrategie verfolgt. Zunächst ist wichtig, Eltern, Kinder und Betreuungspersonen über das Krankheitsbild und mögliche Provokationsfaktoren, die zur Verschlechterung der vorbekannten Symptomatik oder selten zu anaphylaktischen Reaktionen führen können, gut aufzuklären [7].

Eine symptomatische Therapie mit H_1_-Antihistaminika ist Mittel der Wahl bei Pruritus, Flushing, Quaddel- und Blasenbildung [16]. Wie oben erwähnt, zeigt ein Großteil der Kinder mit einer Mastozytose keine oder nur geringe Mediatorsymptome, so dass meist eine Bedarfstherapie mit Antihistaminika ausreicht. Nur selten ist bei ausgeprägter Beschwerdesymptomatik eine Dauermedikation mit Antihistaminika erforderlich [9, 16].

In den meisten Fällen reicht eine Bedarfstherapie mit Antihistaminika aus

Eine UV-Therapie sollte bei Kindern aufgrund der Nebenwirkungen nur in Ausnahmefällen eingesetzt werden. H_2_-Antihistaminika, Cromoglicinsäure oder Protonenpumpeninhibitoren zeigen z. T. gute Wirkung bei gastrointestinalen Beschwerden.

Die gewichtsadaptierte Verordnung eines Notfallsets mit einem Adrenalin-Fertigpen zur i.m.-Injektion, H_1_-Antihistaminikum in flüssiger Form oder als Schmelztablette sowie einem Kortikoidsuppositorium wird für Kinder mit ausgeprägtem Hautbefall, Anaphylaxie oder schweren Symptomen in der Vorgeschichte oder hohem Tryptasewert empfohlen. Kinder, Eltern und enges Umfeld sollten in der Anwendung des Adrenalin-Pens geschult werden. Auch ist die Verordnung eines zweiten Notfall-Sets für Kindergarten oder Schule sinnvoll. Für Kinder mit weniger als 7,5 kgKG ist kein Fertiginjektor erhältlich. Im Notfall sollte bei diesen Kindern die gewichtsadaptierte i.m.-Applikation von 0,01 mg Adrenalin/kgKG erfolgen [4, 16].

Nach jeder Impfung wird eine Überwachung von ca. 1–2 h angeraten

Impfungen werden bei Mastozytose meist gut toleriert und führen nur selten zu kurzfristigen Mediatorsymptomen wie Juckreiz, Quaddeln, Erythem, Flush oder Hypotonie. Eine Prämedikation mit einem H_1_-Antihistaminikum kann erwogen werden. Nach der Impfung wird eine Überwachung von ca. 1–2 h angeraten. Bei diffuser kutaner Beteiligung oder ausgeprägten Mediatorsymptomen wird die Impfung mit nicht mehr als einem Impfstoff pro Konsultation empfohlen [13, 16].

Im Fall von anstehenden Narkosen ist es wichtig, die behandelnden Ärztinnen und Ärzte über die Mastozytoseerkrankung und Reaktionen in der Vorgeschichte aufzuklären. Um mögliche mastzellvermittelte Reaktionen während des Eingriffs zu minimieren, wird eine Prämedikation mit einem H_1_-Antihistaminikum 1–2 h vor dem Eingriff empfohlen. Für längere Narkosen kann eine begleitende Antihistaminikabehandlung über mehrere Tage vor und nach der Narkose erfolgen.

Mastozytome bilden sich meist im Laufe der Zeit von allein zurück. Bestehen Beschwerden wie Blasenbildung, starker Juckreiz und rezidivierende Flush-Symptomatik, kann ein topisches Kortikoid, möglicherweise auch okklusiv, verabreicht werden. Eine Exzision des Mastozytoms sollte nur in Ausnahmefällen bei starkem Leidensdruck und ungünstiger Lokalisation diskutiert werden [16].

Eine Wirkstoffgruppe, die auch bei anderen Erkrankungen mit *KIT*-Mutation, wie GIST, zum Einsatz kommt, sind die Tyrosinkinaseinhibitoren. Bei zwei Kindern mit schwerer Symptomatik bei diffuser kutaner Mastozytose und bestehender *KIT*-Mutation außerhalb von Exon 17 sowie fehlendem Ansprechen auf die symptomatische Therapie konnte Imatinib erfolgreich verabreicht werden [20]. Jedoch sollte Imatinib in der Pädiatrie sehr zurückhaltend und nur mit strenger Indikationsstellung verordnet werden, da es unter der Therapie zu Wachstumsverzögerungen kommen kann [16]. Imatinib wirkt nicht bei Patientinnen und Patienten mit einer *KIT*-D816V-Mutation. Für die adulte fortgeschrittene systemische Mastozytose wurden die KIT-spezifischen Tyrosinkinaseinhibitoren Midostaurin und Avapritinib entwickelt, die auch bei *KIT*-D816V-Mutation wirken [8]. In Einzelfallberichten sprachen auch schwer betroffene Kinder mit einer systemischer Mastozytose auf Midostaurin an [14, 18].

## Fazit für die Praxis

- Die Mastozytose im Kindesalter ist ein seltenes und heterogenes Krankheitsbild, das im überwiegenden Teil der Fälle durch einen gutartigen Verlauf gekennzeichnet ist.
- Das Darier-Zeichen ist pathognomonisch für die Mastozytose und gilt als diagnostischer Marker.
- Mastzellmediatorsymptome wie Juckreiz, Quaddel- und Blasenbildung sowie Flushing können bei der Mastozytose durch verschiedene Trigger provoziert werden. Kinder, Eltern und nahes Umfeld sollten über das Erkrankungsbild und die Trigger-Faktoren aufgeklärt werden.
- Die symptomatische Therapie der ersten Wahl erfolgt mit nichtsedierenden H_1_-Antihistaminika. Abhängig von der Beschwerdehäufigkeit werden sie als Bedarfs- oder Dauermedikation verordnet.
- Ein Notfallset mit Adrenalin-Autoinjektor ist bei ausgeprägtem Hautbefall, hohen Tryptasewerten und schweren Mastzellmediatorsymptomen oder Anaphylaxie in der Vorgeschichte zu verordnen.
- Impfungen sollten bei Kindern mit Mastozytose nach den Empfehlungen der Ständigen Impfkommission (STIKO) durchgeführt werden. Nur in Einzelfällen ist eine Abwandlung des Impfregimes notwendig. Nach der Impfung sollte eine Beobachtungszeit von 1–2 h eingehalten werden.
